# The effect of provincial policies on seismic disaster mitigation in China: An empirical study

**DOI:** 10.1371/journal.pone.0306867

**Published:** 2024-07-09

**Authors:** Lei Gao, Xiaoxue Liu, Yiming Zhao, Longjie Chen, Yihua Que, Zining Wang

**Affiliations:** 1 School of Economics and Management, Institute of Disaster Prevention, Sanhe, China; 2 Department of Disciplines and Graduate Studies, Institute of Disaster Prevention, Sanhe, China; University of L’Aquila: Universita degli Studi dell’Aquila, ITALY

## Abstract

With the development of earthquake disaster reduction efforts in China, the content of earthquake disaster reduction policies has become increasingly enriched and improved. Particularly, multiple provincial governments have proposed earthquake disaster reduction planning policies. It is important to explore whether these policies can affect disaster mitigation. Therefore, this paper summarizes the earthquake disaster reduction plans and factors influencing seismic resilience. Panel data from 24 provinces between 2012 and 2021 were collected, and a difference-in-differences approach was used to construct an econometric model to evaluate the policy effects and analyze the enhancement of seismic resilience. The results show that the implementation of earthquake disaster reduction policies has a positive impact on earthquake monitoring, evacuation, and emergency relief capabilities, and the estimated policy effects are statistically significant. Moreover, a series of tests were conducted. The conclusions are as follows: (1) Earthquake disaster reduction policies have a positive impact on the improvement of seismic resilience in provinces. (2) Provinces with a higher number of earthquakes experience more significant effects from earthquake disaster reduction policies. (3) Provinces with higher seismic peak ground acceleration values exhibit more pronounced improvements in seismic resilience.

## Introduction

China is one of the countries with the most severe natural disasters in the world, characterized by a wide variety of disasters, widespread geographical distribution, and high occurrence frequency. Among them, earthquakes are one of the major disasters [1]. Historical earthquakes in China have caused enormous loss of life and property. In 1976, the 7.8-magnitude Tangshan earthquake and the 7.1-magnitude Luanxian earthquake in Hebei Province resulted in severe destruction due to their occurrence in densely populated areas. In 2008, the 8.0-magnitude Wenchuan earthquake in Sichuan Province was the largest and most destructive earthquake in China since its founding, causing significant loss of life and property. In 2022, a 6.8-magnitude earthquake occurred in Luding County, Sichuan Province, which prompted the initiation of a Level 2 earthquake emergency response by the Sichuan Provincial Earthquake Administration.

According to the "*2022 National Natural Disaster Overview*" published by the Ministry of Emergency Management, earthquakes were one of the major natural disasters in China in 2022, with relatively active earthquakes in the western regions and significant disaster losses [2]. Additionally, based on data from the China Statistical Yearbook, the direct economic losses attributable to earthquake disasters in the past five years have amounted to 40 billion CNY.

As a result, to enhance the capacity for earthquake disaster prevention, establish a seismic disaster prevention and mitigation mechanism, and minimize earthquake disaster losses, China Earthquake Administration has been conducting research on the development planning of national seismic disaster prevention and mitigation since 2007. In 2012, the "*National 12th Five-Year Plan for Seismic Disaster Prevention and Mitigation*" was released, which included eight plans for seismic disaster prevention and mitigation, including monitoring planning, defense planning, legal construction planning, emergency rescue planning, informatization planning, and publicity planning [3–8]. Detailed arrangements were made for monitoring, prevention, and rescue measures for earthquake disasters. In 2016, the "*Seismic Disaster Prevention and Mitigation Plan (2016–2020)*" was issued, which made comprehensive arrangements for earthquake disaster response and post-disaster reconstruction from aspects such as earthquake monitoring and early warning, seismic defense capabilities, emergency rescue capabilities, institutional construction, and technological support [9]. Since then, 15 provinces, including Shaanxi, Gansu, Shanxi, Guangxi, Henan, and Inner Mongolia, have successively formulated seismic disaster prevention and mitigation plans during the "13th Five-Year Plan" period, providing more detailed planning for each province. In 2020, China Earthquake Administration issued the "*China Seismic Station Network Plan (2020–2030)*", "*China Geophysical Station Network (Geomagnetic) Plan*", "*China Geophysical Station Network (Gravity) Plan*", and "*China Geophysical Station Network (Crustal Deformation) Plan*" [10–13]. Various planning policies propose solutions to improve China’s seismic monitoring capacity. In 2022, the State Council issued the "*14th Five-Year Plan for National Emergency Response System*", the Ministry of Emergency Management and China Earthquake Administration jointly issued the "*14th Five-Year Plan for National Seismic Disaster Prevention and Mitigation*" [1, 14]. Similarly, these two policies summarize the achievements in seismic disaster prevention and mitigation during the "13th Five-Year Plan" period and outline the development direction for the "14th Five-Year Plan" period.

With the developing of national seismic disaster prevention and mitigation efforts, the content of seismic disaster prevention and mitigation planning has become increasingly comprehensive. Meanwhile, there are also various factors to consider in planning policies. Therefore, it is necessary to investigate the implementation effect of seismic disaster prevention and mitigation planning. Does it have a significant impact on the seismic disaster prevention and mitigation capabilities of various provinces? A thorough study of these questions will contribute to a comprehensive and scientific evaluation of the implementation effect of seismic disaster prevention and mitigation planning. Accordingly, this paper focuses on evaluating the effectiveness of earthquake prevention and disaster reduction policies at the provincial level, specifically targeting 13 provinces in China that have implemented plans for more than five years during the 13th Five-Year Plan period. Key elements suitable for evaluating the effectiveness of these policies are identified, including seismic monitoring capability, earthquake evacuation capacity, and emergency rescue capability. Moreover, a difference-in-differences model will be used to assess the impact of seismic disaster prevention and mitigation planning on the provincial level. This study will fill this gap in research on the policy effects of earthquake prevention and mitigation in China. Findings will provide useful references to implementation of seismic mitigation planning in other countries.

The remaining sections of this paper are as follows: In the literature review section, we describe the current research status of seismic disaster prevention and mitigation capabilities and the study of policy effects, reviewing previous research content and methods. The materials and methods section presents the research design, including model construction, experimental data, and variable explanations. The empirical results section provides an analysis of the basic results of the experiment, robustness tests, and heterogeneity analysis. The discussion section provides an extended discussion of this study. The conclusions section presents the conclusions of the paper.

## Literature review

### Different stages of seismic disaster

Before an earthquake disaster occurs, numerous studies focus on monitoring technologies. After the Wenchuan earthquake in 2008, China Earthquake Administration strengthened earthquake monitoring and forecasting [15]. In the past decade, seismic networks have significantly improved in terms of coverage density, data quality, and instrumental diversity [16]. According to data from China Statistical Yearbook, China currently has over 6,500 seismic stations and more than 35,800 macroscopic observation points. Therefore, the seismic data has also become more diverse and detailed. In terms of earthquake prediction, current mainly involve studying the fundamental principles and laws of earthquakes [17], the total gradient of gravity level [18], and earthquake precursors [19]. These studies have made certain predictions for earthquakes in certain regions, effectively reducing casualties and property losses. Therefore, the improvement of seismic monitoring and early warning capabilities has a positive impact on urban seismic disaster prevention and mitigation capabilities.

Similarly, seismic monitoring and warning technologies also have an influence on the evacuation during an earthquake disaster. Factors under seismic conditions can affect the time required for evacuation, such as panic among individuals and coverage of disaster damage [20]. The improvement of monitoring and warning technologies can provide earlier warning, which in turn facilitates the evacuation of more people [21]. Meanwhile, the suddenness of earthquake disasters can affect individuals’ decision-making abilities in various ways [22]. Therefore, the planning of evacuation routes in densely populated areas is an important part of personnel evacuation. A well-designed route can effectively enhance the speed of personnel evacuation, meaning that in the event of an earthquake, dense crowds can evacuate in an orderly manner, reducing or even avoiding casualties [23]. As a result, this impacts urban seismic disaster prevention and mitigation capabilities positively.

After the occurrence of an earthquake disaster, one of the important aspects is the emergency response. The disaster prevention and mitigation are mainly reflected in emergency response systems, transportation capabilities, and medical system rescue capabilities. Furthermore, the earthquake emergency response system can provide various seismic parameters for preliminary assessment and valuable information for emergency rescue [24]. Meanwhile, during the rescue process, the improvement of urban transportation capabilities can effectively avoid congestion on rescue routes and timely transport the injured to hospitals. Therefore, establishing a resilient transportation network with medical facilities along the routes is crucial [25]. Moreover, after transporting injured individuals to hospitals, their treatment becomes a top priority in disaster relief work, and the development of rescue capabilities of urban medical systems is significant [26]. Therefore, improving urban emergency response capabilities helps enhance post-earthquake rescue efficiency, reducing casualties and economic losses, and ultimately enhancing urban seismic disaster prevention and mitigation capabilities.

### Policy effectiveness

In order to enhance the seismic disaster prevention and mitigation capabilities of various provinces in China, many provinces have issued the seismic disaster prevention and mitigation plans. The plans not only include the earthquake monitoring capabilities, evacuation capabilities and emergency relief capabilities above mentioned, but also provide detailed contents for other earthquake prevention and mitigation capabilities. As a result, this study intends to investigate the impact of seismic disaster prevention and mitigation plans on the capabilities of each province in China to evaluate their implementation effects.

In research on the implementation effects of policies, there are currently three main methods. The first is the entropy weight method [27]. This method quantitatively evaluates the weights of various indicators to calculate the policy index for each city, thereby assessing the degree to which policy objectives are achieved. However, the disadvantage of entropy weight method is that it cannot consider the influence of non-policy factors on policy. The second method is breakpoint regression analysis. It assesses policy effects by determining whether the dependent variable exhibits a significant breakpoint in the year of policy implementation. If there is a clear breakpoint before and after policy implementation, it indicates that the policy has a significant impact on the dependent variable [28]. However, using breakpoint regression analysis may lead to underfitting when building regression models, thus affecting accuracy. The third method is the difference-in-differences analysis (DID). This method divides the sample provinces into experimental and control groups, considering both the differences in the dependent variable before and after policy implementation and the differences in the dependent variable between the experimental and control groups. For instance, Zhang Ji et al. [29] evaluated the effects of the coordinated development policy in the Beijing-Tianjin-Hebei region on household economic risks using a difference-in-differences model. In addition, in the research of the policy implementation effect, other scholars have used methods such as the whole process evaluation based on comparative perspective and TVP state space equation to evaluate the implementation effectiveness of different policies [30, 31]. Due to the impact of the provincial policies includes various aspects, these aspects are coordinated and developed to improve China’s resilience to earthquake prevention and disaster reduction. Meanwhile, DID model can take into account the differences before and after policy implementation, as well as the differences in different provinces, so that the effectiveness of policy implementation can be more clearly demonstrated. Therefore, the DID approach is more valuable for research in the period of policy transition of various areas. For example, the following application areas have been recorded in the literature: DID model study the impact and mechanisms of high-speed rail service intensity on CO2 emissions [32], examine the effects of electricity price subsidies policy on NOX emissions and removal [33], study the effects of low-carbon city pilot policies in China [34], investigate the impact of carbon emissions trading system in China [35], study the impact and mechanisms of high-speed rail on urban land expansion [36].

### Research gaps and contributions

In summary, prior studies have primarily concentrated on enhancing earthquake prevention and mitigation capacities, with a predominant focus on technical aspects. However, there is a dearth of assessment the implementation effects of earthquake prevention and disaster reduction planning. Furthermore, there is a lack of in-depth analysis regarding the robustness and heterogeneity of policy implementation. Therefore, this study aims to investigate the contents, tasks, and methods proposed in the "13th Five-Year Plan" for seismic disaster prevention and mitigation, select the difference-in-differences model, identify the factors influencing provincial seismic disaster prevention and mitigation capabilities, and study the implementation effects of seismic disaster prevention and mitigation planning policies. The analysis considers three main factors contributing to the effectiveness of policy elements in earthquake prevention and disaster reduction: seismic monitoring, earthquake evacuation, and emergency rescue. Finally, policy recommendations will be proposed to enhance seismic disaster prevention and mitigation capabilities.

## Materials and methods

**Fig 1:**
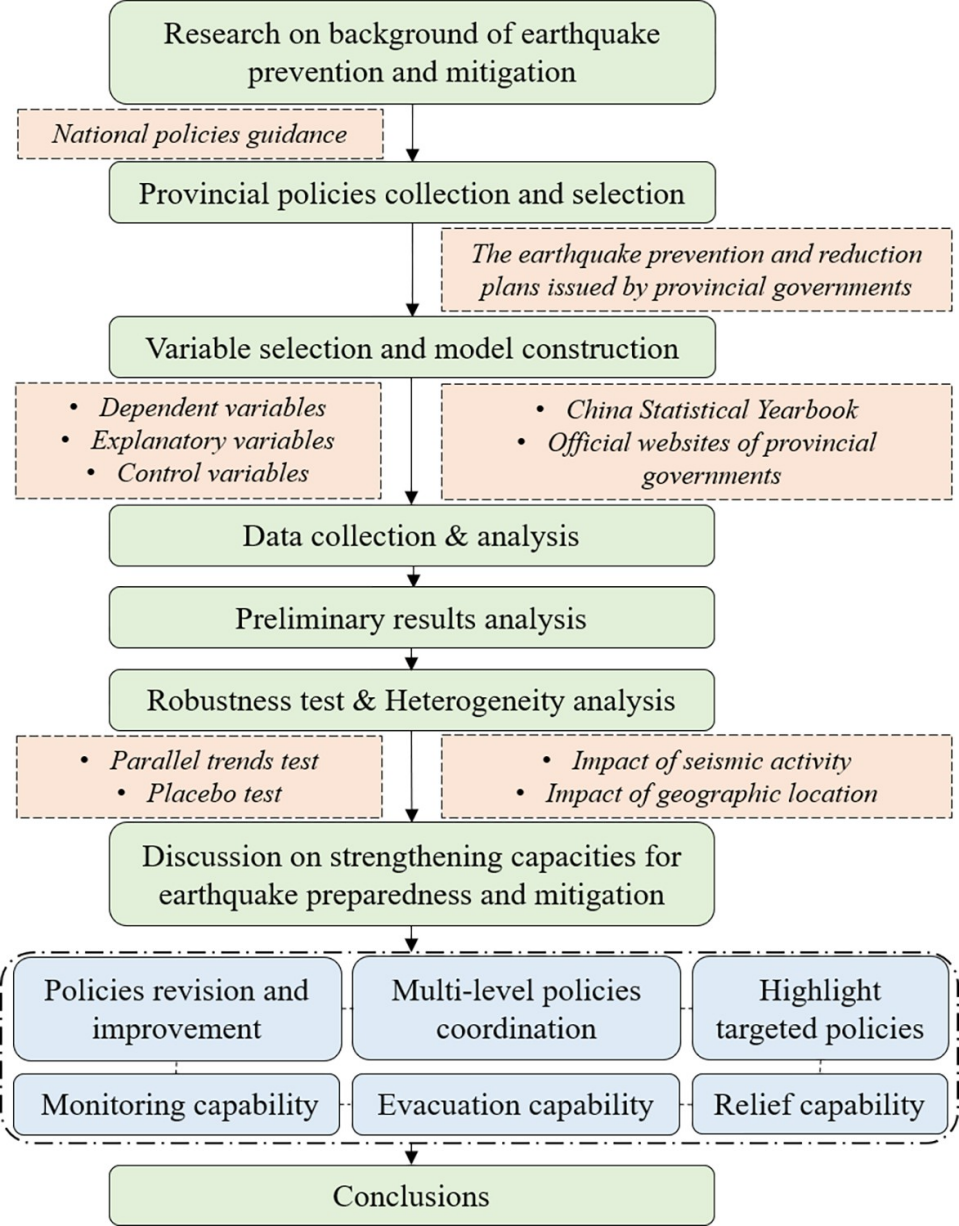
Research procedure and methods.

The research procedure and methods in this paper are shown in Fig 1.

This study used SPSSAU and Stata MP 17 software for data processing, analysis, and testing. SPSSAU is a web-based data analysis platform widely used in humanities, social sciences, natural sciences, and other disciplines. In this study, it was primarily used for data analysis. Stata is a comprehensive statistical software that provides functions for data analysis, data management, and generating professional charts. In this study, Stata was mainly used for results testing. Finally, based on the experimental results, the influence of provincial seismic disaster prevention and mitigation planning on seismic mitigation capabilities was analyzed.

### Sample selection and data sources

**Fig 2:**
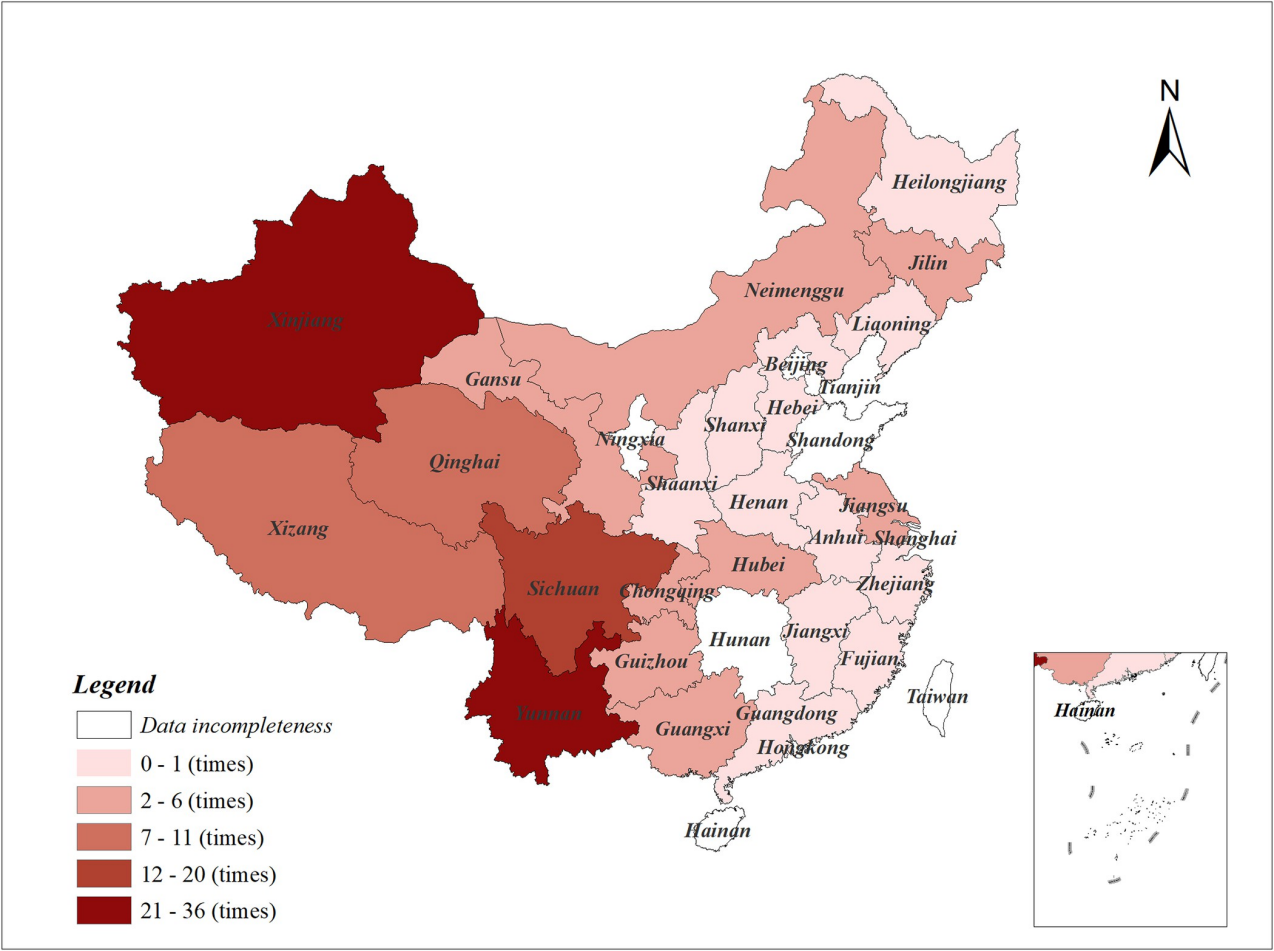
The frequency of earthquakes in each province of China from 2012 to 2021 (sourced from China Statistical Yearbook and drawn by the authors).

**Fig 3:**
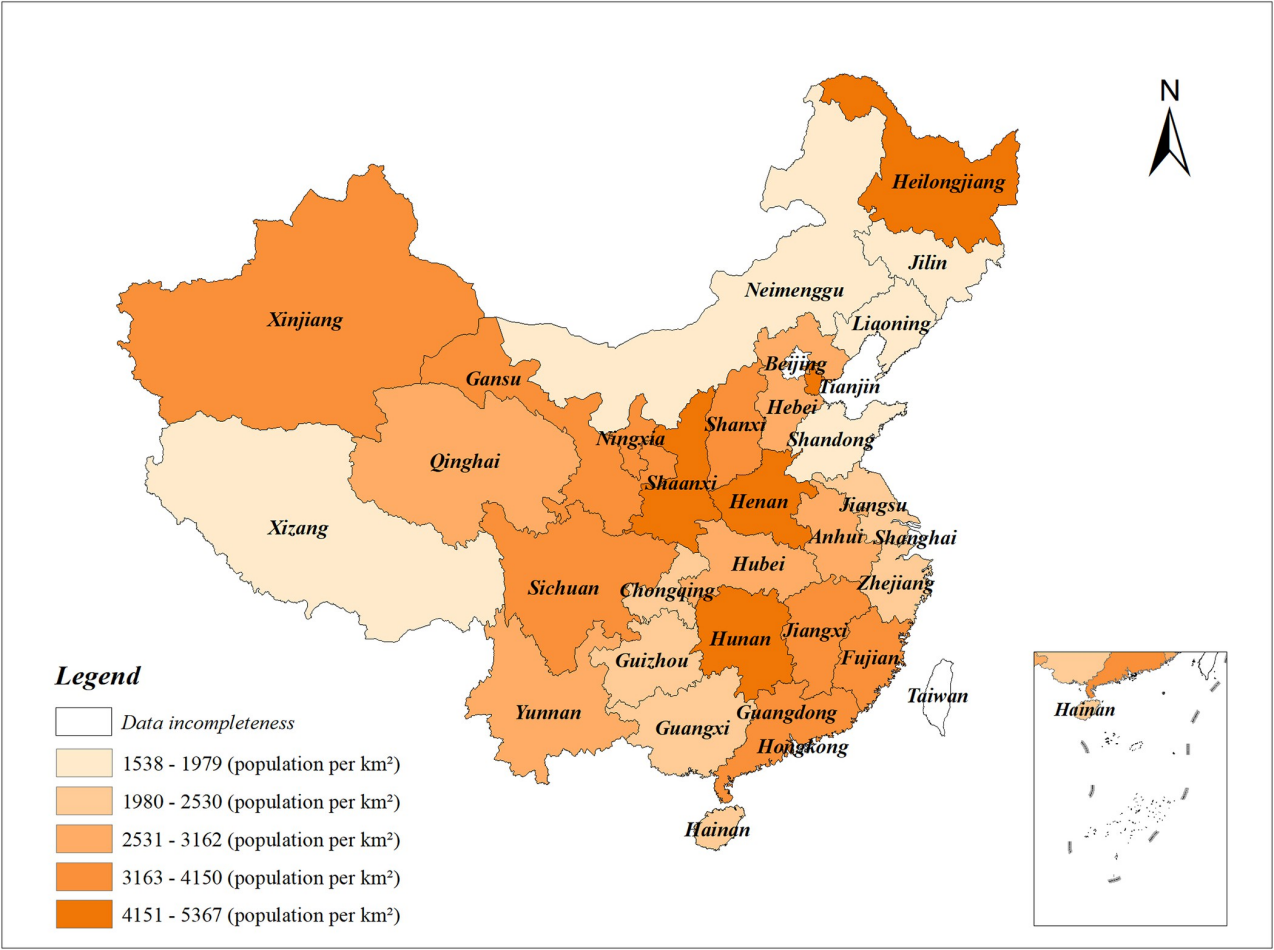
Urban population density of China’s provinces in 2021 (sourced from China Statistical Yearbook and drawn by the authors).

This study was carried out on the basis of provincial data related to earthquake hazards from 2012 to 2021. Historical data on earthquake disasters demonstrate that areas with higher population densities are more severely affected by earthquakes compared to areas with lower population densities. On the one hand, the western region of China had a higher number of earthquakes during the period of 2012–2021, indicating a higher level of seismic activity in the western region (Fig 2). On the other hand, Fig 3 illustrates the distribution of urban population densities within Chinese provinces in 2021, the population in China is mainly concentrated in the central region. Therefore, the risk of casualties in the central region is higher than in other regions following an earthquake. The provincial policies on earthquake prevention and mitigation should be given more attention in both central and western regions.

**Fig 4:**
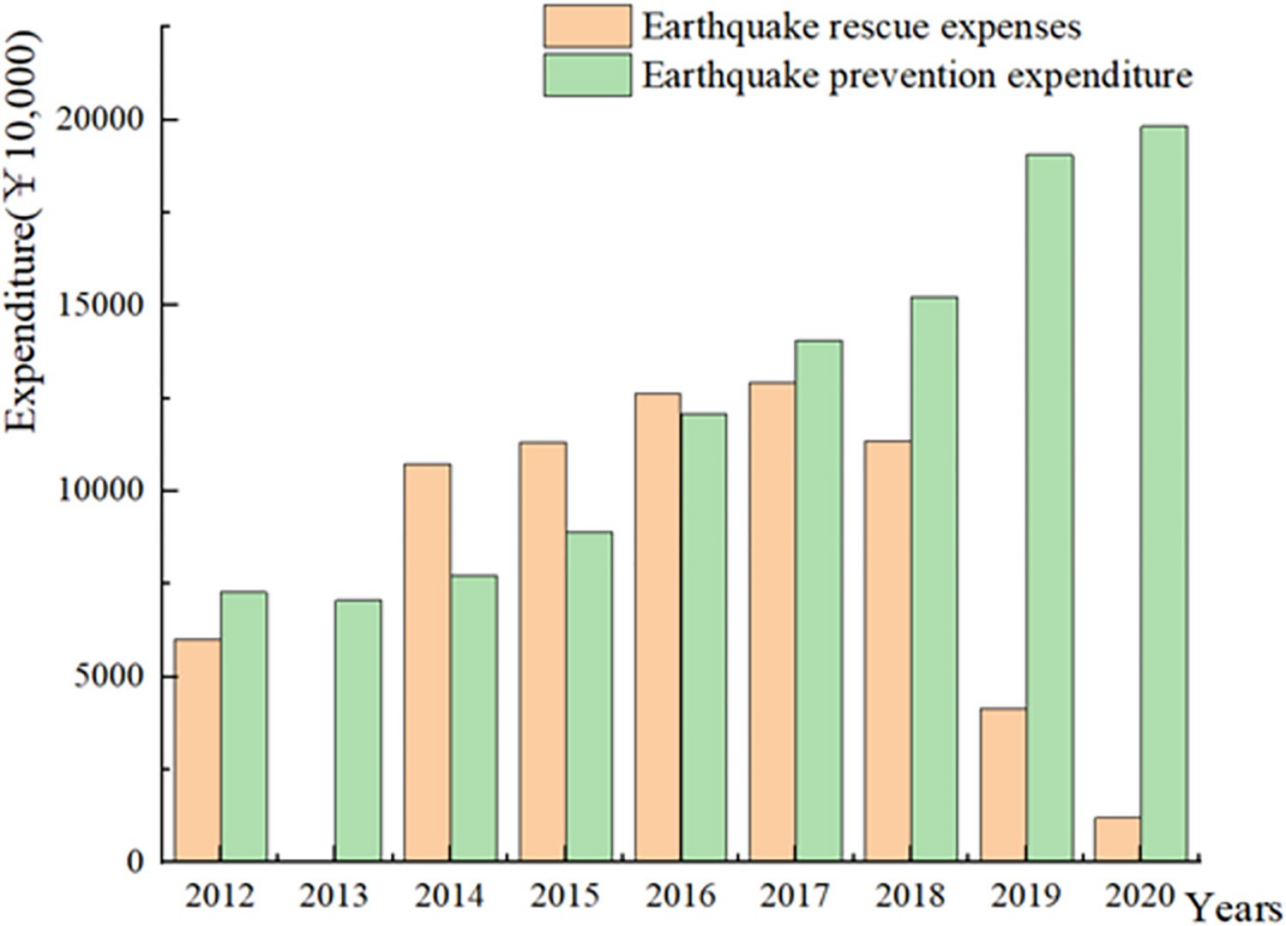
China’s earthquake prevention and rescue expenditures from 2012 to 2020.

**Fig 5:**
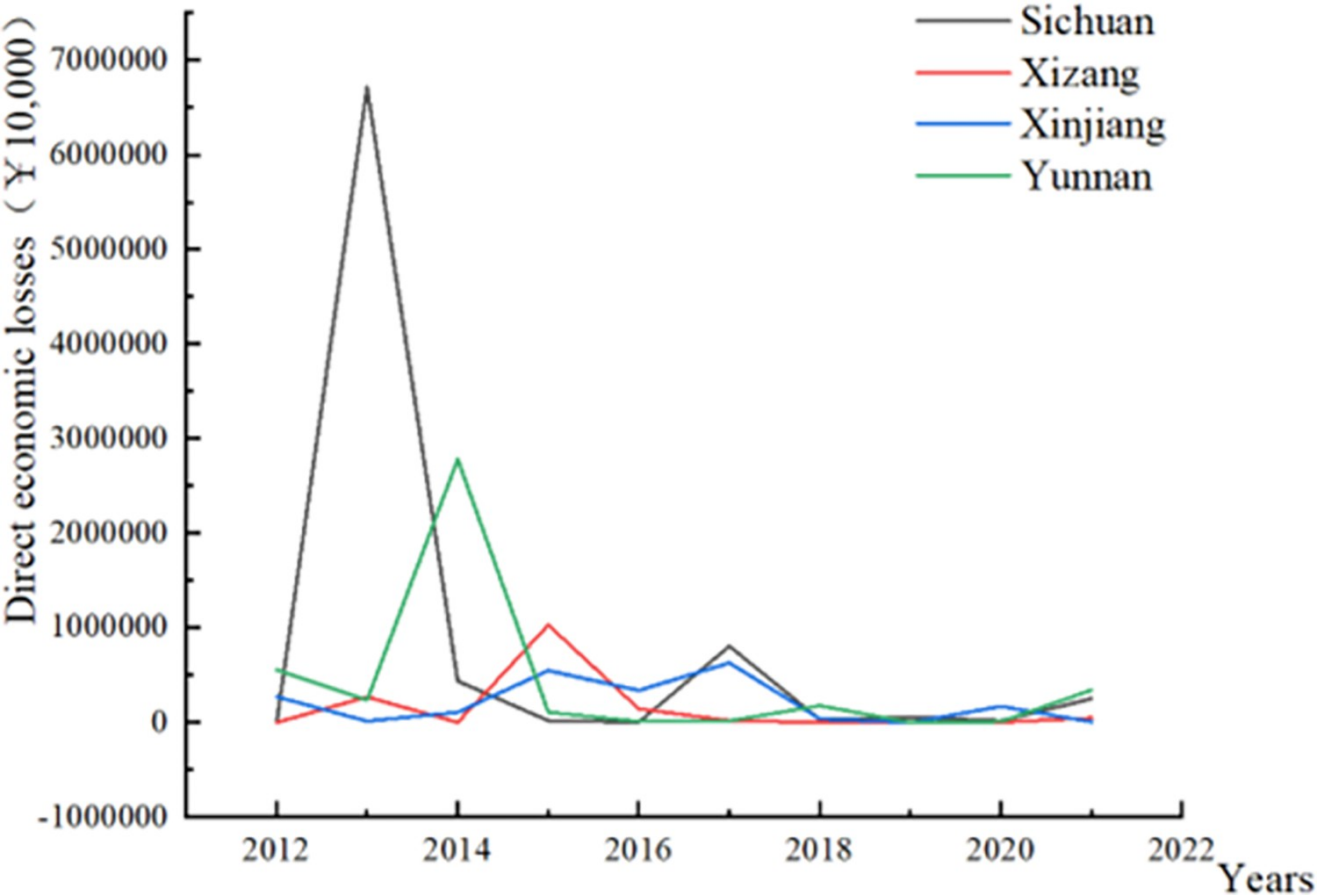
Direct economic losses caused by seismic activity in the past decade (Taking Sichuan, Tibet, Xinjiang, and Yunnan provinces as examples).

From the perspective of financial expenditures of 2012 to 2020, earthquake rescue expenditures were higher than earthquake prevention expenditures before 2016. However, after 2016, earthquake prevention expenditures gradually increased and surpassed rescue expenditures (Fig 4). This indicates a shift in national investment towards earthquake prevention efforts since 2016, aiming to reduce casualties and property losses by prioritizing prevention work. Additionally, for the four provinces with active seismicity, there is a downward trend in direct economic losses caused by earthquakes (Fig 5). This trend is likely the result of a decrease in the frequency or magnitude of earthquakes. However, the level of economic losses is also influenced to some extent by the country’s economic support for infrastructure construction in various provinces. With increased investment in earthquake prevention work over the past decade, there have been little direct economic losses since 2016. Similarly, the "*National 13th Five-Year Plan for seismic disaster prevention and mitigation*" also emphasizes the enhancement of seismic resistance capacity of infrastructure, such as rural houses, schools, hospitals and old buildings in cities [9]. Since the implementation of the "13th Five-Year Plan" in 2016, with the reinforcement of building seismic resistance, expenditures on earthquake rescue activities have gradually decreased. This also indicates the progressive enhancement of the nation’s seismic resistance capabilities.

Therefore, based on the above data characteristics related to earthquake hazards, several essential factors must be addressed when selecting samples and data for assessing earthquake mitigation capacity, such as the frequency of earthquakes, the risk of casualty losses, prevention capacity, and expenditure on prevention and rescue.

This study is intended to explore the implementation effectiveness of earthquake prevention and mitigation policies. The data were sourced from the "China Statistical Yearbook" from 2012 to 2021, and provincial planning policies of earthquake disaster reduction was obtained from the official websites of China Earthquake Administration, Ministry of Emergency Management, Ministry of Finance and Provincial Earthquake Administration. By searching and summarizing the earthquake disaster prevention and mitigation plans of each province, it can be found that 13 central and western provinces released the "13th Five-Year Plan" policies for earthquake prevention and disaster reduction. Accordingly, panel data on the factors influencing seismic disaster prevention and mitigation capabilities of 24 provinces from 2012 to 2021 were constructed for analysis. The 13 provinces that released the provincial-level plan were selected as the experimental group, while the 11 provinces that did not release the plan served as the control group. In addition, the implementation of the earthquake disaster prevention and mitigation planning policies during the "13th Five-Year Plan" period began in 2016. Therefore, the policy effects are mainly demonstrated in the post-2016 period.

### Model assumptions

In order to facilitate the construction and solutions of the model and make them match the actual situation, some assumptions and simplifications need to be made to the problem:

1. Due to the characteristics of differences in issuance timing of the planning policies in a few provinces, it is possible that some of the contents of planning were already pilot-tested during the preparation phase before the official release. Therefore, the policies identified in this study were all published around 2016, assuming that the effects of the 13th Five-Year Plan policies occurred between 2016 and 2020. Similarly, this implies that the treatment groups received their treatments at approximately the same time or very close time.
2. National-level planning has an equal guiding effect on all provinces. Furthermore, this study does not take into account any specific investments or funding from the national level for earthquake disaster reduction in a particular province.
3. Provinces and municipalities that have issued earthquake disaster reduction planning will be influenced by this planning policy in various phases: before, during, and after a disaster. Meanwhile, the provinces in the control group that did not issue their own planning policies, regardless of the reasons for their failure to issue the planning policies, were not affected by the planning policies during the study planning period.

### Mathematical description of the model

In this paper, the difference-in-differences approach is used to assess the implementation effects of seismic disaster reduction planning policies and their impact on the provincial disaster mitigation capacity. The difference-in-differences method evaluates policy effects by comparing the differences between the experimental group and the control group before and after policy implementation, and analyzes the influencing factors. Therefore, the sample provinces were divided into an experimental group and a control group based on whether they implemented the seismic disaster reduction plans. The model for assessing the impact of planning policies on seismic resilience is as follows:

$$Y_{it}=\alpha_{0}+\alpha_{1}Treat_{i}*Period_{t}+\alpha_{2}X_{it}+\beta_{i}+\gamma_{t}+\varepsilon_{it}$$ (1)

Where i represents the province, t represents the year, Y_it_ is the dependent variable, which represents the seismic resilience of city i at time t. Treat_i_ is a dummy variable indicating whether the province implemented the seismic disaster reduction plans, and Period_t_ is a dummy variable representing the publication time of the plans. Meanwhile, the interaction term Treat_i_*Period_t_ denotes the change in seismic resilience of the experimental group after policy implementation, with the coefficient α_1_ measuring the effects of the earthquake disaster reduction plans. Furthermore, X_it_ represents the control variables that affect seismic resilience, including three parts: seismic characteristics, seismic losses, urban population. β_i_ denotes province fixed effects controlling for factors that do not vary over time at the provincial level, γ_t_ represents time fixed effects controlling for factors that do not vary across regions over time, and ε_it_ denotes the error term.

### Variables and data description

#### Selection of dependent variables

This study primarily investigates the impact of provincial-level earthquake disaster reduction planning policies. According to the earthquake prevention and mitigation plan (2016–2020) issued by National Development and Reform Commission and China Earthquake Administration [9], many provinces in China have issued their earthquake prevention and mitigation plans. The 13th Five-Year Plan collection is shown in Table 1.

**Table 1:** The seismic disaster prevention and reduction policies of 13 provinces.

Provinces	Document types	Issue by
Xinjiang	13th Five-Year Integrated Plan for Disaster Prevention and Reduction [37]	PPG
Guangdong	13th Five Year Plan for Earthquake Prevention and Disaster Reduction [38]	PDRC, PEA
Sichuan	13th Five Year Plan for Earthquake Prevention and Disaster Reduction [39]	PDRC, PEA
Chongqing	13th Five Year Plan for Earthquake Prevention and Disaster Reduction [40]	PDRC, PEA
Yunnan	13th Five Year Plan for Earthquake Prevention and Disaster Reduction [41]	PDRC, PEA
Hubei	13th Five Year Plan for Earthquake Prevention and Disaster Reduction [42]	PDRC, PEA
Jiangxi	13th Five Year Plan for Earthquake Prevention and Disaster Reduction [43]	PDRC, PEA
Anhui	13th Five Year Plan for Earthquake Prevention and Disaster Reduction [44]	PPG
Inner Mongolia	13th Five Year Plan for Earthquake Prevention and Disaster Reduction [45]	PDRC, PEA
Henan	13th Five Year Plan for Earthquake Prevention and Disaster Reduction [46]	PPG
Guangxi	13th Five Year Plan for Earthquake Prevention and Disaster Reduction [47]	PDRC, PEA
Shanxi	13th Five-Year Integrated Plan for Disaster Prevention and Reduction [48]	PDRC, PEA
Gansu	13th Five Year Plan for Earthquake Prevention and Disaster Reduction [49]	PPG

PPG: Provincial People’s Government; PDRC: Provincial Development and Reform Commission; PEA: Provincial Earthquake Agency.

**Fig 6:**
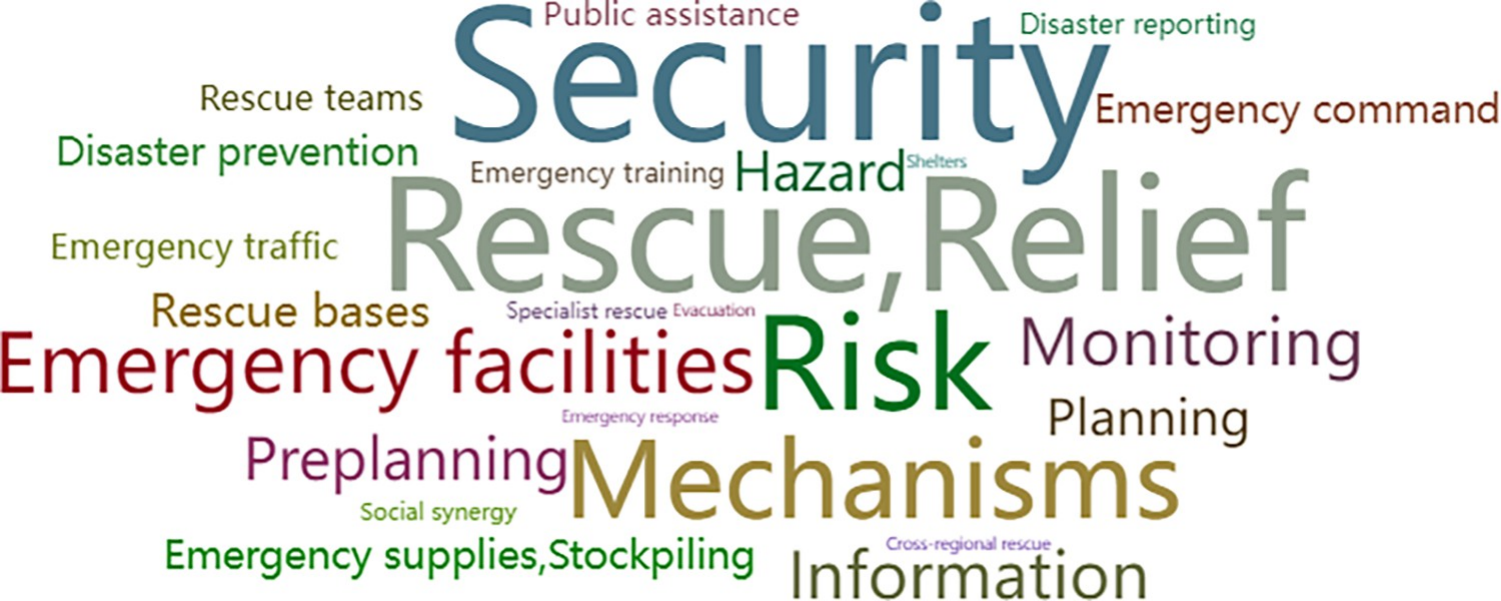
Cloud map of high-frequency words in the policy texts of national government. (Note: Sourced from national policies) [1, 9, 14].

**Fig 7:**
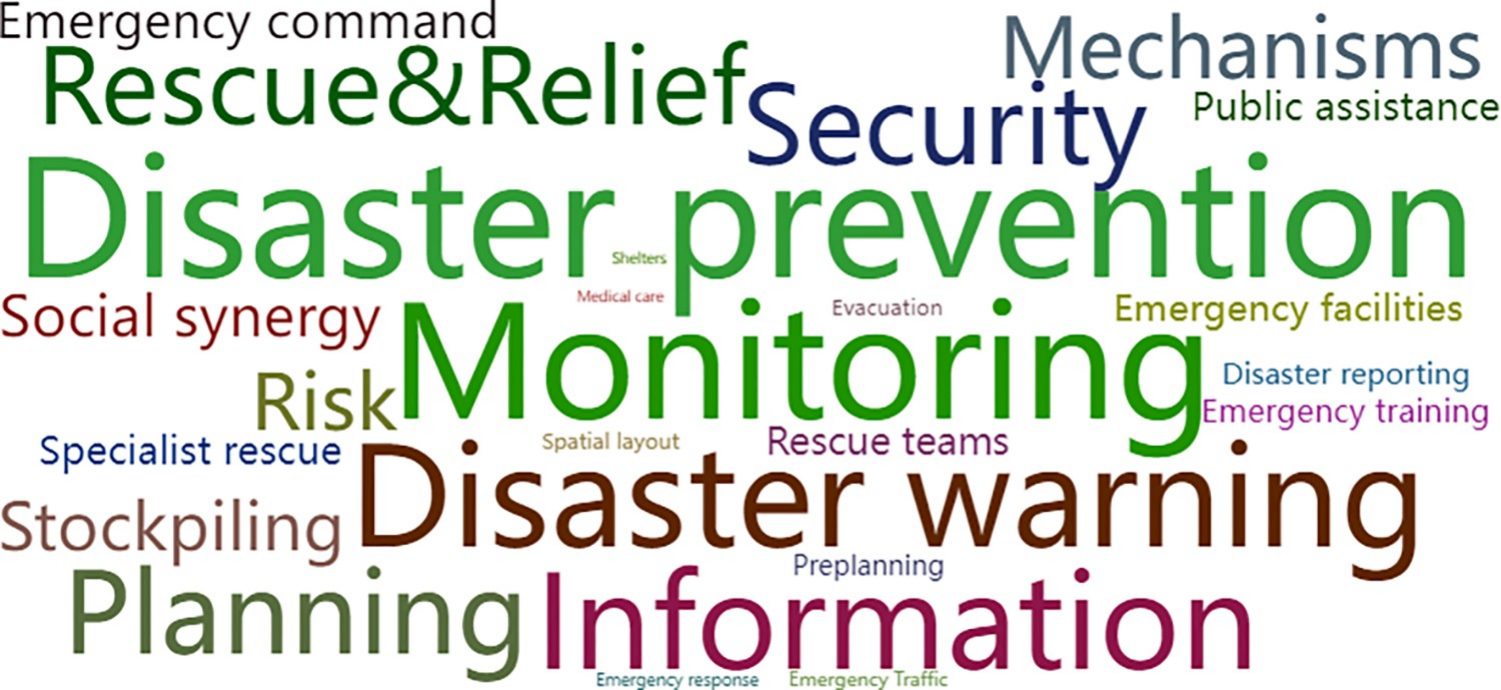
Cloud map of high-frequency words in the policy texts of provincial governments. (Note: Sourced from provincial policies) [37–49].

Policies issued by national and provincial governments were used to study the key factors of plan policies of earthquake prevention and disaster reduction. Using the policy text analysis method, we built a policy text database on the basis of 3 national and 13 provincial policies to conduct the analysis. In this paper, based on the term frequency analysis tool, the policy texts were word-sorted and high-frequency words were counted. Furthermore, in order to identify the high-frequency words, we selected the top 25 words and built a vocabulary cloud map, as shown in Figs 6 and 7. The size of the words in the cloud is determined by the frequency of the words, and the higher the frequency, the larger the font size. We can see that "Disaster prevention", "Information", "Mechanisms", "Monitoring", "Rescue & Relief", "Risk", "Security", "Emergency command", "Emergency facilities" and various key words are all in the core words of the national and provincial governments.

Therefore, in the process of variable selection, frequency analysis of words is an important reference. Based on the research described above, we found that the composition of vocabulary reflects four logically connected elements faced during earthquake disasters, which include disaster preparedness, disaster monitoring, disaster evacuation, and disaster relief.

Firstly, in the stage of disaster preparedness, the content includes constructing a defensive system mechanism, which includes key words such as "Mechanism", "Planning", "Security", and "Preplanning". Secondly, the content of earthquake disaster monitoring includes key words such as "Disaster reporting", "Monitoring", and "Disaster warning". Thirdly, emergency evacuation during a disaster includes key words such as "Emergency command", "Emergency facilities", "Emergency traffic", "Medical care". Fourthly, earthquake relief includes key words such as "Specialist rescue", "Rescue teams", "Relief".

On this basis, we conduct semantic analysis and classification, for example, assigning "Information", "Risk" to the key elements of earthquake disaster monitoring, and assigning "Emergency response", "Public assistance", "Social synergy" to the key elements of earthquake relief.

Accordingly, based on the focus points of the literature review section and the word frequency analysis mentioned above, and considering data availability, the earthquake monitoring capacity, earthquake evacuation capacity, and emergency relief capacity were selected to represent the comprehensive seismic resilience of provinces [50].

1. Earthquake monitoring capacity
  Earthquake monitoring capacity is mainly reflected in the investment of seismic monitoring services. This implication means that the more investment there is in seismic monitoring services, the more importance the province attaches to its seismic monitoring capacity. Therefore, this study uses expenditures on earthquake monitoring affairs (Monitor) as a proxy for earthquake monitoring capacity, and the natural logarithm of the variable is used in the analysis.
2. Earthquake evacuation capacity
  Earthquake evacuation capacity is mainly reflected in the selection and planning of evacuation sites. The availability of open spaces, such as squares and vacant land, affects the selection of evacuation sites. Therefore, the more emptiness areas there are, the more choices there are for refuge places. This paper uses the per capita park green space area (Area) to express the earthquake evacuation capacity.
3. Emergency relief capacity
  Emergency medical care and the transportation of relief supplies are crucial aspects of earthquake emergency relief capacity. Adequate medical personnel are essential for the treatment of injured individuals. Therefore, this study uses the number of medical personnel per thousand people (Medical) as a proxy for medical emergency relief capacity. Similarly, the transportation of relief supplies after an earthquake also affects post-disaster recovery. A higher proportion of land allocated for transportation infrastructure indicates higher transportation efficiency for relief supplies. Therefore, this study uses the ratio of transportation land use (Tran) (transportation land area/total urban area) as a proxy for transportation emergency relief capacity.

#### Core explanatory variables

This study constructs a dummy variable to indicate whether provinces implemented earthquake disaster reduction plans. Therefore, it is divided into an experimental group and a control group. An experimental group consists of 13 provinces where the plans were implemented and issued by China Earthquake Administration. These provinces are Xinjiang, Guangdong, Sichuan, Chongqing, Yunnan, Hubei, Jiangxi, Anhui, Inner Mongolia, Henan, Guangxi, Shanxi, and Gansu. The basic information of the plans is presented in Table 1. The remaining 11 provinces serve as the control group, including Jilin, Jiangsu, Hebei, Zhejiang, Fujian, Tibet, Guizhou, Liaoning, Shaanxi, Qinghai, and Heilongjiang. In the experimental group, Treat_i_ is set to 1, while in the control group, Treat_i_ is set to 0. The dummy variable Period_t_ represents the publication time of the earthquake disaster reduction plans. If the sample province is in or after 2016, Period_t_ is assigned a value of 1; otherwise, it is assigned a value of 0. The core explanatory variable in this study is the interaction term Treat_i_ * Period_t_, which indicates whether province i implemented earthquake disaster reduction plans at time t.

#### Control variables

In order to accurately assess the impact of earthquake disaster reduction plans on seismic mitigation capacity at the provincial level, it is important to avoid other factors that may affect seismic resilience. Since the varying seismic conditions across provinces, this study selects earthquake frequency (Earthnum), the number of casualties in earthquake disasters (Percas), the number of deaths in earthquake disasters (Perdie), direct economic losses from earthquake disasters (Eartheco), and urban population density (Perden) within the province as control variables.

Earthquake frequency (Earthnum) reflects the level of seismic activity and its impact on earthquake disaster reduction efforts in a province. A higher frequency indicates more severe challenges in earthquake disaster reduction.

The number of casualties (Percas) and deaths (Perdie) in earthquake disasters reflect the psychological impact on affected individuals. Provinces with higher casualties and deaths are more likely to have a stronger determination to enhance earthquake disaster reduction efforts.

Direct economic losses (Eartheco) from earthquake disasters indicate the impact on the provincial economy, with greater losses having a larger influence on future earthquake disaster reduction efforts.

Urban population density (Perden) within the province affects the extent of losses and casualties following earthquakes. Provinces with higher population density require more time for evacuation, increasing the risk of casualties.

Descriptive statistics of these variables are presented in Table 2.

**Table 2:** Descriptive statistics of key variables.

Variable type	Variable name	Variable symbol	Meaning of variables	Number of samples	Average	Standard deviation	Min	Max
Dependent variables	Earthquake monitoring capacity	Monitor	Logarithmic value of economic inputs for seismic monitoring	240	241.31	445.76	0	3305.57
	Earthquake evacuation capacity	Area	Park space per capita	240	12.953	2.963	2.36	19.96
	Emergency relief capacity	Medical	Number of medical personnel per 1,000 population	240	6.251	1.265	3.03	9.32
		Tran	Transport land use rate	240	6.649	7.125	0.02	34.06
Explanatory variable	Dummy variables of policy effects	Treat*Period	Whether a seismic mitigation plan has been issued	240	0.325	0.469	0	1
Control variables	Seismic characteristics	Earthnum	Frequency of seismic disasters	240	0.567	1.289	0	8
	Seismic Losses	Percas	Casualties from earthquake disasters	240	1569	15702	0	229000
		Perdie	Death toll from earthquake disasters	240	4.596	42.592	0	618
		Eartheco	Direct Economic Losses in Seismic Disasters	240	82080	504898	0	6714639
	Urban population	Perden	Urban population density in provinces	240	2981.8	1111.4	1032	5541

## Empirical results

### Descriptive statistical analysis

According to Table 2, descriptive statistical analysis does not prove that changes in provincial seismic mitigation capacity are due to the implementation of seismic mitigation planning. Additionally, it cannot eliminate the possibility of other factors influencing the results. Therefore, to further investigate the relationship between the changes in seismic resilience and the implementation of these plans, a difference-in-differences approach is used.

### Preliminary results analysis

Based on a quasi-natural experiment sample consisting of 13 provinces that implemented earthquake disaster reduction plans between 2012 and 2021, a difference-in-differences model is utilized to analyze the impact of these plans. The preliminary results are presented in Table 3. It can be clearly seen that the implementation of earthquake disaster reduction plans significantly improved the seismic resilience of provinces during 13th Five-Year Plan period. Similarly, the improvements of earthquake monitoring capacity, earthquake evacuation capacity, and emergency relief capacity can be witnessed.

**Table 3:** Preliminary results.

	Monitor		Area			Medical		Tran	
	(1)	(2)	(3)	(4)	(5)	(6)	(7)	(8)	(9)
Treat×Period	0.914*** (3.951)	1.241*** (6.330)	1.495*** (2.698)	1.093** (2.595)	1.472* (1.772)	0.664*** (2.841)	1.576*** (14.171)	3.762*** (2.642)	6.996*** (7.761)
Earthnum	0.031 (0.668)	0.033 (0.365)	-0.541*** (-3.242)	0.130 (1.011)	-0.566*** (-3.219)	0.071 (1.091)	0.097* (1.723)	-1.470*** (-5.191)	-0.173 (-0.441)
Percas	-0.000*** (-2.612)	-0.000** (-2.428)	0.000 (1.430)	0.000*** (4.141)	0.000 (0.383)	-0.000*** (-4.305)	-0.000*** (-2.848)	-0.000 (-0.764)	0.000 (0.472)
Perdie	-0.001** (-2.589)	-0.001 (-1.486)	0.005** (2.506)	-0.000 (-0.084)	0.005** (2.304)	-0.003*** (-3.301)	-0.002*** (-2.792)	0.014*** (4.150)	0.001 (0.210)
Eartheco	-0.000** (-2.433)	-0.000 (-1.383)	-0.000*** (-3.008)	0.000 (1.215)	-0.000** (-2.166)	-0.000** (-2.062)	-0.000** (-2.585)	-0.000*** (-3.348)	0.000 (1.015)
Perden	-0.000 (-0.247)	-0.000 (-0.124)	-0.000 (-1.120)	0.000 (0.825)	-0.000 (-1.202)	-0.000 (-0.289)	-0.000 (-0.183)	-0.000 (-0.434)	0.002 (1.299)
Time fixed effects	No	No	No	No	Yes	No	No	No	No
Individual fixed effects	No	Yes	No	Yes	No	No	Yes	No	Yes
Sample sizes	240	240	240	240	240	240	240	240	240
*R* ^2^	0.146	0.157	0.106	0.047	0.105	0.216	0.318	0.122	0.209

Note: * *p*<0.1

** *p*<0.05

*** *p*<0.01, t-value is in ()

Specifically, the results of Models (1)-(2) demonstrate a positive effect of the implementation of earthquake disaster reduction plans on earthquake monitoring capacity. The OLS regression estimates and fixed effects estimates indicate coefficients of 0.914 and 1.241, respectively, both statistically significant at the 1% level.

Models (3)-(5) investigate the impact of these plans on earthquake evacuation capacity among provinces. The results indicate that the implementation of earthquake disaster reduction plans significantly enhances earthquake evacuation capacity. The OLS regression estimates and policy effects estimates with different levels of fixed effects are 1.495, 1.093, and 1.472, respectively, all statistically significant at the 1%, 5%, and 10% levels.

Models (6)-(9) examine the influence of earthquake disaster reduction plans on emergency relief capacity. The results demonstrate a positive effect of these plans on emergency relief capacity. The OLS regression estimates for the number of medical personnel per thousand people (Medical) are 0.664 and 3.762, respectively, both statistically significant at the 1% level. Similarly, the ratio of transportation land use (Tran) corresponding fixed effects estimates are 1.576 and 6.996, respectively, also statistically significant at the 1% level.

In summary, the preliminary results indicate that the regression results of the policy effects dummy variables are consistently positive. This suggests that provincial-level earthquake disaster reduction plans effectively improve earthquake monitoring capacity, earthquake evacuation capacity, and emergency relief capacity, consequently enhancing the provincial integrated disaster reduction capacity.

### Robustness test

#### Parallel trends test

The parallel trend test is a correlation test to assess whether there is some kind of increase or decrease of the same magnitude between the data of two variables. In other words, it compares the data points before and after the publication of the earthquake mitigation plans to determine if there are significant changes. The parallel trends test is a prerequisite for the difference-in-differences approach. Therefore, the use of the difference-in-differences method requires that the dependent variable for both the treatment and control groups show parallel trends before the release of the plans.

In this study, a t-test is conducted to examine the parallel trends of the dependent variables. The results are presented in Tables 4 and 5. Table 4 shows that before the implementation of earthquake disaster reduction plans, the p-values of t-test for the dependent variables Monitor, Area, Medical, and Tran are all greater than 0.1 for both the experimental and control groups. This indicates that there are no significant differences between the two groups, which satisfies the parallel trends assumption and enables the adoption of the difference-in-differences model.

**Table 4:** T-test (Before).

Explanatory variables	Control groups (*n* = 44)	Treated groups (*n* = 52)	*p*
Monitor	1.519	1.781	0.230
Area	12.074	12.744	0.130
Medical	5.400	5.132	0.141
Tran	2.324	1.756	0.435

*p<0.1

**p<0.05

***p<0.01

**Table 5:** T-test (After).

Explanatory variables	Control groups (*n* = 66)	Treated groups (*n* = 78)	*p*
Monitor	7.130	6.733	0.022**
Area	12.691	13.808	0.043**
Medical	0.207	0.515	0.091*
Tran	10.690	8.931	0.158

*p<0.1

**p<0.05

***p<0.01

As it is shown in Table 5, after the implementation of earthquake disaster reduction plans, significant differences are observed in the experimental group compared to the control group in terms of Monitor, Area, and Medical. However, there is no significant difference in Tran although it exhibits a positive effect. This suggests that the implementation of earthquake disaster reduction plans has a positive impact on the integrated seismic resilience of provinces. The lack of significant differences in Tran may be due to a lag in the effects of earthquake disaster reduction policies on the construction of transportation infrastructure.

#### Placebo test

To further ensure the accuracy of the experimental results, a placebo test is conducted using a counterfactual approach [51]. The 13 provinces are assigned as the pseudo-experimental group, while the remaining 11 provinces serve as the pseudo-control group. Pseudo-policy dummy variables are generated to test the effects of implementing earthquake disaster reduction plans, yielding estimates of the pseudo-policy effects. If the estimates of the pseudo-policy effects are not statistically significant, it indicates that the improvement in seismic monitoring capacity, earthquake evacuation capacity, and emergency relief capacity is indeed a result of the plans’ implementation and not due to other random factors. This would indicate robustness in the conclusions.

**Fig 8:**
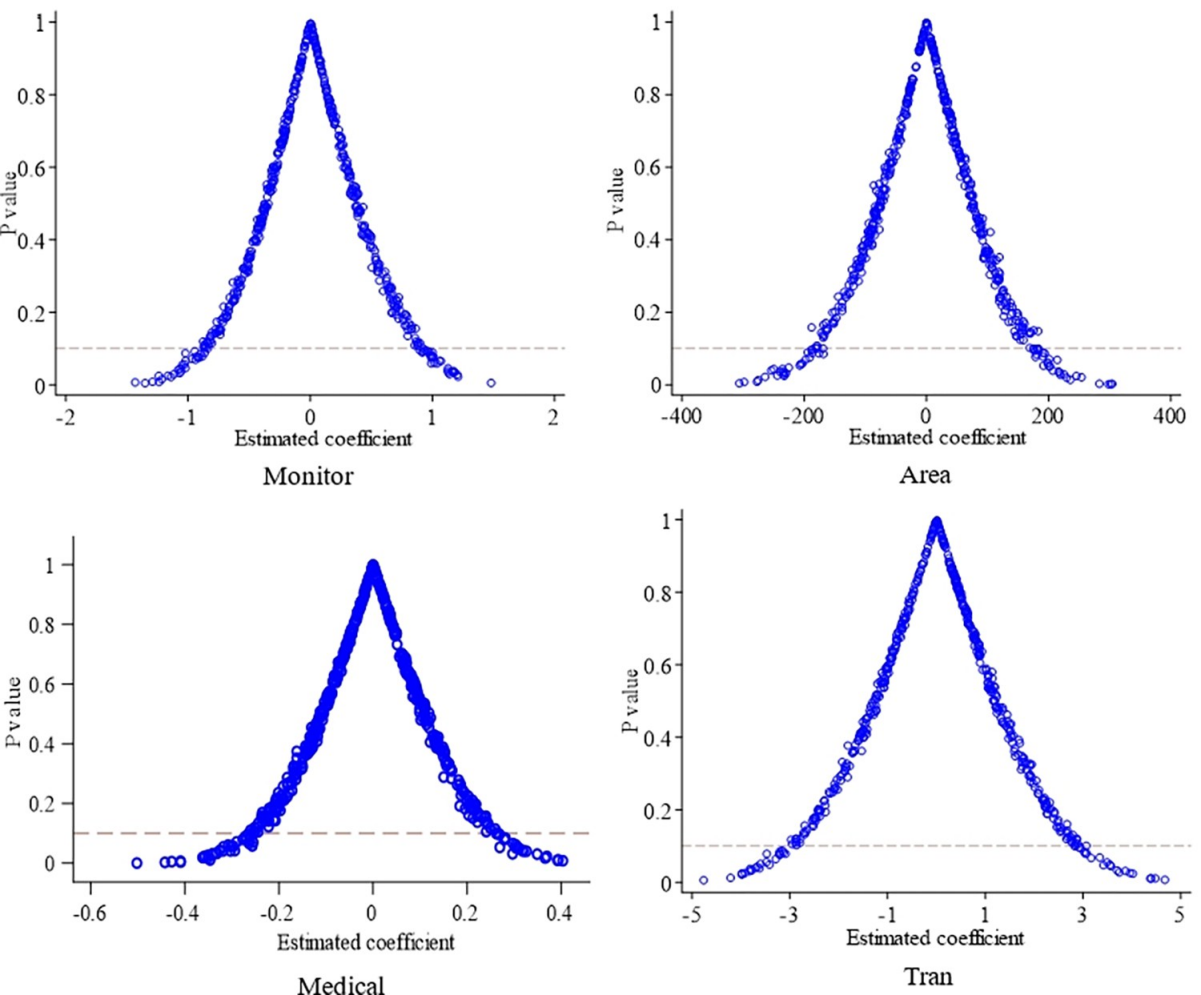
Placebo test results.

Along these lines of placebo test, Fig 8 displays the distribution of estimated pseudo-policy effects after conducting the placebo test 500 times. The x-axis represents the estimated policy effects, while the y-axis represents the corresponding p-values. The dashed horizontal line represents the 10% significance level with a p-value of 0.1. It can be observed that after artificially creating the experimental group, the estimated coefficients for the four dependent variables (Monitor, Area, Medical, Tran) have p-values mostly exceeding 0.1 at the 10% significance level. This suggests that the estimates obtained in this study are not random. Consequently, the improvement in seismic monitoring capacity, earthquake evacuation capacity, and emergency relief capacity among provinces that implemented earthquake disaster reduction plans can be attributed to the implementation of these plans, rather than other policies or random factors.

### Heterogeneity analysis

#### The impact of seismic activity patterns on the effectiveness of earthquake disaster reduction plans

Due to the variations in seismicity in different regions, the effectiveness of earthquake disaster reduction plans may differ. In this study, the sample provinces are divided into three regions based on the number of earthquakes occurring within a decade: less than 2, 2–6, and more than 6 times. Baseline regressions are conducted for each of these regions, and the empirical results are presented in Table 6. The study finds that the policy effects are more prominent in all three regions in terms of improving earthquake monitoring capacity. Regarding the improvement in earthquake evacuation capacity, the policy effects are significant for regions with less than 2 earthquakes, but not significant for provinces with 2–6 earthquakes or more than 6 earthquakes. For the enhancement of medical and emergency relief capacities, the policy effects are significant for provinces with 2–6 earthquakes and more than 6 earthquakes. In terms of improving transportation capacity, the policy effects are significant for provinces with less than 2 earthquakes and more than 6 earthquakes, but not significant for provinces with 2–6 earthquakes.

**Table 6:** Heterogeneity results based on earthquake frequency.

Frequency of earthquakes	Variables	Monitor	Area	Medical	Tran
<2	Treat×Period	0.671** (2.342)	2.019*** (5.079)	0.123 (0.436)	6.771*** (4.825)
	Control variables	Control	Control	Control	Control
	N	240	240	240	240
	R^2^	0.079	0.233	0.004	0.211
2–6	Treat×Period	0.641** (2.090)	1.143 (1.481)	1.109*** (4.262)	1.333 (0.700)
	Control variables	Control	Control	Control	Control
	N	240	240	240	240
	R^2^	0.134	0.163	0.271	0.037
>6	Treat×Period	1.959*** (6.444)	1.138 (1.258)	1.064*** (2.881)	3.780*** (4.661)
	Control variables	Control	Control	Control	Control
	N	240	240	240	240
	R^2^	0.512	0.104	0.316	0.372

*p<0.1

**p<0.05

***p<0.01

The reasons behind these findings lie in the fact that provinces with fewer than 2 earthquakes experience fewer losses caused by seismic events due to their lower occurrence. Consequently, these regions prioritize the improvement of earthquake monitoring and shelter capacities. On the other hand, provinces with more than 6 earthquakes face frequent seismic activities. Enhancing emergency relief capabilities in these regions helps mitigate the various losses associated with earthquakes. Therefore, these provinces place greater emphasis on improving earthquake emergency relief capacities.

#### The impact of geographic location on the implementation effectiveness of earthquake disaster reduction

The variations in earthquake occurrence probabilities and intensities due to geographical differences. Therefore, this study divides the provinces based on *“China’s Seismic Ground Motion Peak Acceleration Zoning Map (GB18306-2015)”* [52]. According to the publication GB18306-2015, areas with a peak ground motion acceleration exceeding 0.3g are considered to have a higher probability of earthquakes, as they are located within potential seismic sources. As a result, a dummy variable "Geo" is created, with a value of 1 for provinces in regions with a peak ground motion acceleration exceeding 0.3g and 0 for other provinces. The estimation equation for the model is as follows:

$$Y_{it}=\alpha_{0}+\delta Treat_{i}\times Period_{t}\times Geo+\beta_{1}Treat_{i}\times Period_{t}+\beta_{2}Treat_{i}\times Geo+\beta_{3}Period_{t}\times Geo+\gamma X_{it}+\varepsilon_{it}$$ (2)

Where i represents the province, t represents the year, Y_it_ denote the dependent variable representing the seismic resilience of province i at time t. Treat_i_ is a dummy variable indicating whether the province implemented seismic disaster reduction plans, and Period_t_ is a dummy variable representing the publication time of the plans. Geo is a dummy variable representing the peak ground motion acceleration. The interaction term Treat_i_× Period_t_× Geo denotes the change in seismic resilience in areas with a peak ground motion acceleration exceeding 0.3g after policy implementation, with the coefficient δ measuring the effects of the earthquake disaster reduction plans. Additionally, X represents the control variables affecting seismic resilience, while εit denotes the error term.

The estimation results of the model are presented in Table 7. Obviously, it can be observed that all three interaction terms have significantly positive coefficients, indicating that the higher the probability of earthquake occurrence in a province, the greater the impact of earthquake disaster reduction plans on enhancing seismic resilience.

**Table 7:** Heterogeneity results based on geographic location.

	Monitor	Area	Medical	Tran
Treat×Period×Geo	0.702*** (3.175)	1.184** (2.132)	1.712*** (6.237)	4.123*** (5.290)
Control Variables	Control	Control	Control	Control
Time fixed effects	Yes	No	No	No
Individual fixed effects	Yes	Yes	Yes	Yes
*R* ^2^	0.060	0.024	0.127	0.052
Sample sizes	240	240	240	240

*p<0.1

**p<0.05

***p<0.01, t-value is in ()

Accordingly, the results of the heterogeneity analysis demonstrate that the implementation effectiveness of provincial-level earthquake disaster reduction plans is influenced by earthquake conditions and geographic location. Different regions prioritize different aspects of earthquake disaster reduction plans. Provinces with a higher probability and intensity of earthquakes exhibit better implementation effectiveness of the planning policies.

The reasons behind these findings are as follows: Provinces located in seismically active areas pay more attention to the improvement of earthquake monitoring, evacuation, and emergency relief capacities compared to other regions. Therefore, when formulating earthquake disaster reduction plans, greater emphasis is placed on enhancing seismic resilience. For example, the *"13th Five-Year Plan for Earthquake Disaster Reduction in Sichuan Province"* emphasizes the strengthening of earthquake monitoring, prediction, warning capabilities, earthquake disaster defense capabilities, and earthquake emergency response capabilities. It includes projects such as earthquake intensity rapid reporting and early warning engineering, the Sichuan-Yunnan national earthquake monitoring and prediction experimental field, and the integration of earthquake disaster risk defense and emergency response engineering [39]. Similarly, provinces that have experienced major earthquakes often prioritize the resistance of buildings to withstand significant seismic events. These provinces are more psychologically affected by earthquake disasters, and thus, they have higher requirements for the seismic resistance of buildings to minimize the impact on people’s lives and mental well-being.

## Discussion

According to this study, earthquake mitigation planning policies will directly promote the improvement of provincial seismic resilience in China. From an international perspective, despite the diversity of stages of industrialization and urbanization, the model of promulgation of planning policies for disaster prevention has similar applicability between China and other countries. Furthermore, several topical issues on how to better enhance earthquake prevention and mitigation capacity are as follows.

1. In recent years, with the gradual improvement of the earthquake prevention and disaster reduction management system, several Chinese departments have jointly or independently issued earthquake prevention and disaster reduction plans, such as Development and Reform Commission, Emergency Management Bureau, Earthquake Agency. The implementation of these plans involves disaster prevention, forecasting, evacuation, rescue and other factors. However, the departments responsible for plan formulation find it difficult to conduct field research and assessment for each implementation. Similarly, evaluating the effectiveness of policy implementation is also a challenge faced by most countries after the promulgation of policies. To address this challenge, it is necessary to make use of a variety of methods to evaluate the implementation effects, identify the utility and potential deficiencies, so that it can gain a better understanding of policy effectiveness, and make targeted revisions and improvements to each plan to achieve implementation effectiveness.
2. In addition to provincial governments, many municipal governments have also issued earthquake disaster reduction plans in China. Especially the higher provincial government where the city is located has already issued an earthquake prevention and mitigation plan. For example, Yichang, Fuyang, and Zhengzhou are located in Hubei, Anhui, and Henan provinces, respectively, and based on the provincial planning, the municipal level has also issued their earthquake mitigation planning [53–55]. However, the policies issued by some cities are simply responses to provincial and national policies. These cities may not have fully considered their own characteristics due to the framework of higher-level policies. Therefore, this research can provide reference for policy formulation in these cities, enabling municipal governments to fully consider factors that influence the improvement of seismic resilience when formulating policies. Additionally, based on this research, the assessment methodology can be applied to the evaluation of multi-administrative level policies both at home and abroad, and help identify and address potential issues in the implementation of the policies.
3. The enhancement of seismic resilience requires improvements in various aspects, including seismic monitoring, evacuation, and emergency relief capabilities. However, our research has revealed that different regions face varying degrees of influence from factors such as earthquake frequency, geographic location, and population demographics, making it difficult to simultaneously improve seismic monitoring, evacuation, and emergency relief capabilities in all regions. This also indicates that there are some disparities and deficiencies in achieving the multifaceted objectives of the policies. Therefore, future policy formulation can focus on identifying the weaknesses in seismic resilience in each province and city, and analyze the spatiotemporal distribution characteristics of common disasters. This will help to develop targeted and comprehensive plans to strengthen resilience and direct limited resources in disaster prevention to areas with the greatest need.
4. From the perspective of earthquake monitoring capability, timely analysis and summarization of disaster situations, as well as the formulation of emergency response measures and the issuance of information to relevant departments and the public, are particularly important for monitoring capabilities. When formulating seismic resilience policies, it is essential to consider how to promptly obtain disaster information, collect disaster intelligence, assess disaster development trends and impact areas. Thus, it is necessary to identify potential disaster threats before they occur and to track the evolution of disasters in real-time after they happen. This will enhance emergency decision-making and command capabilities, thereby improving the disaster prevention capabilities. Additionally, under the backdrop of digital development, it is important to strengthen the construction of digital disaster prevention infrastructure, such as establishing comprehensive management systems for basic information management, ensuring earthquake routine affairs management, and integrating emergency decision-making and command systems. For instance, "one-network management" models can be employed to enhance the efficiency of using basic information.
5. From the perspective of earthquake evacuation capability, the demand for places of refuge should be a primary consideration in seismic prevention and mitigation planning. Based on population distribution, it is necessary to plan and construct sufficient and safe evacuation shelters. Meanwhile, it is necessary to take into account the ability to reach the appropriate places of refuge in timely and secure channels. On one hand, stationary evacuation sites such as city center parks can serve as disaster prevention places. On the other hand, emergency evacuation sites like school playgrounds can also be utilized. Moreover, improving earthquake evacuation capability may involve upgrading seismic evacuation command facilities and emergency medical rescue facilities in existing infrastructure conditions, as well as long-term seismic living facilities, material warehouses and medical treatment facilities. Aims to provide the evacuees with necessities such as food, water, light, tents, medicine and health facilities.
6. From the perspective of emergency relief capability, the formulation of policies to enhance emergency relief capability requires focusing on the four aspects: Firstly, the creation of professional rescue team is essential. This involves enhancing the construction of comprehensive rescue teams, integrating resources, establishing rapid mobilization mechanisms, reinforcing standardized construction of rescue personnel allocation, equipment provisioning, routine training, and logistical support to improve the capability for emergency disaster response. Secondly, it is important to enrich disaster prevention at the grassroots level. This mainly includes integrating firefighting, militia, public security, and other forces, establishing comprehensive emergency response teams in townships. Thirdly, the social participation mechanisms are critical to emergency relief. It is crucial to strongly support the development of social emergency relief teams and perfect the mechanism for government purchasing of emergency relief services. Fourthly, public awareness of emergency disaster response should be raised. It is essential to popularize various types of safety and emergency shelter knowledge, organize diverse forms of public safety knowledge propaganda activities, enhance the awareness of public risk prevention and self-help capabilities, strengthen the social responsibility of enterprises and institutions in carrying out seismic disaster prevention knowledge education and training.

## Conclusions

This paper takes earthquake disaster reduction planning policies as quasi-natural experiments and uses SPSS AU and Stata MP 17 for data processing to empirically examine the impact of earthquake disaster reduction plans on seismic resilience in various provinces. Meanwhile, the difference-in-differences model is suitable for studying the implementation effects of policies. According to parallel trend tests and placebo tests, the accuracy of the basic conclusions is confirmed. Furthermore, this study analyzes the heterogeneity of factors influencing the effectiveness of earthquake disaster reduction plans. In summary, the following conclusions are drawn:

1. Earthquake disaster reduction policies have a positive impact on the improvement of seismic resilience in provinces. Meanwhile, the study finds that positive effects act on earthquake monitoring, evacuation, and emergency relief capabilities. All statistical significance is observed at least at the 1% level. Additionally, the seismic resilience of provinces is also influenced by control variables such as seismic conditions, earthquake losses, and population characteristics.
2. When examining the heterogeneity of seismic activity, provinces with a higher number of earthquakes consistently exhibit p-values less than 0.01. In contrast, other provinces have experienced situations where 0.01 < p < 0.05 or even p > 0.01. This indicates that provinces with a higher number of earthquakes tend to experience more significant effects from earthquake disaster reduction policies. Similarly, provinces with a history of more frequent earthquakes tend to prioritize and develop better earthquake disaster reduction measures.
3. In the analysis of geographical heterogeneity, the inclusion of the dummy variable Geo results in p-values for the regression results that are all statistically significant at least at the 1% level. This suggests that provinces with higher seismic peak ground acceleration values experience greater improvements in seismic resilience. The heterogeneity analysis of earthquake disaster reduction plans reveals that the geographical location of provinces significantly influences the enhancement of seismic resilience.
4. In seismic disaster reduction planning policies, several key contradictions need to be addressed. For instance, there is a gap between the types, reserves, and layout of emergency supplies and the peak demand for responding to catastrophic disasters. The application of new technologies and new techniques is not sufficient, and the comprehensive monitoring and forecasting capabilities for multiple disasters and disaster chains need to be improved. Additionally, the construction of comprehensive disaster laboratories and experimental fields and other research platforms is inadequate. Similarly, the reinforcement of disaster prevention, reduction, and relief forces also requires attention in the future development of seismic disaster reduction planning.
5. From the perspective of policymakers, policymakers should prioritize the sustainability of policy effectiveness, and as the policy implementation period nears its end, they should diligently employ scientific methods to analyze and optimize the subsequent stage of policies. The method presented in this article is not only applicable to policy analysis and evaluation but also serves as a foundation for extending more scientific policy evaluation methods. In addition, the current policy formulation operates within the framework of territorial management. Thus, policymakers must consider the synergy among governments at various levels and across different regions when addressing earthquake disasters. This approach ensures the most efficient and rational implementation of cross-provincial joint forecasting, rescue operations, and other measures.

This study provides the analysis and corresponding tests on the effects of China’s provincial-level planning policies related to seismic reduction. These findings help to understand the policy implementation of provincial seismic reduction in China. Moreover, the formulation of other disaster prevention policies also involves various factors, including disaster supervision, evacuation, and rescue. To identify executable and quantifiable elements suitable for policy implementation, the exploration, analysis, and evaluation process of these research elements can draw upon the findings of this study. Similarly, the findings will also provide useful inputs for implementation of seismic mitigation planning in other countries.

## Supporting information

S1 Table Related data. (XLS)

S2 Table Data of Figs 2 and 3. The frequency of earthquakes in each province of China from 2012 to 2021, and Urban population density of China’s provinces in 2021.
(XLSX)

S3 Table Data of Fig 4. China’s earthquake prevention and rescue expenditures from 2012 to 2020.
(XLS)

S4 Table Data of Fig 5. Direct economic losses caused by seismic activity in the past decade.
(XLSX)
